# Axial rotation of the hinge axis can cause changes in coronal tibial alignment in anterior tibial closing wedge osteotomy in a 3D simulation model

**DOI:** 10.1002/jeo2.70198

**Published:** 2025-03-07

**Authors:** Julius Watrinet, Philipp Blum, Lukas Willinger, Julian Mehl, Sebastian Siebenlist, Markus Bormann, Wolfgang Böcker, Boris Holzapfel, Julian Fürmetz

**Affiliations:** ^1^ Department Trauma Surgery BG Unfallklinik Murnau Murnau German; ^2^ Department of Orthopaedic Sports Medicine, School of Medicine Technical University of Munich Munich Germany; ^3^ Department of Orthopedics and Trauma Surgery, Musculoskeletal University Center Munich (MUM) University Hospital, LMU Munich Munich Germany

**Keywords:** ACWO, osteotomy, posterior tibial slope, simulation

## Abstract

**Purpose:**

Clinical evidence indicates that an unintended increase in the medial proximal tibial angle (MPTA) can occur during slope‐reducing tibial osteotomies, which is most relevant in anterior cruciate ligament (ACL) deficient knees. Therefore, the purpose of this three‐dimensional (3D) simulation study is to assess how axial or coronal hinge axis rotation affect alignment parameters in anterior tibial closing wedge osteotomies (ACWO). The hypothesis states that a neutral hinge axis (NHA) in ACWO prevents changes in coronal and axial alignment.

**Methods:**

A 3D surgical simulation was used to perform ACWO with a stepwise increment of one‐degree (1°–5°) rotation around thirteen different hinge axes. Surface models were created from CT scans of 49 individuals (mean PTS 11.5° ± 3.8°) resulting in 3185 simulations. A NHA not changing the coronal or axial alignment was defined as oriented parallel to the posterior tibial plateau 1 cm underneath the articular surface. Anatomical landmarks were determined for each simulation to measure the PTS, MPTA, hip‐knee‐ankle angle (HKA), and tibial torsion (TT). The effects of the initial MPTA or PTS on the resulting alignment parameters and the effects of axial and sagittal rotation of the joint axis were analysed for their effects on postsimulation PTS, MPTA and TT.

**Results:**

Clinically relevant hinge axis rotation in the coronal or axial plane below 20° did not significantly influence PTS correction (*p* > 0.05). Axial rotation of the hinge axis exceeding 10° led to significant MPTA changes (> +1.7° ± 0.03°) compared to the NHA (*p* < 0.001). Pre‐simulation MPTA had no influence on post‐simulation MPTA changes (*p* > 0.05).

**Conclusion:**

NHA aligned parallel to the posterior tibial plateau below the articular surface prevents significant changes in MPTA during ATCWO. This 3D simulation suggest, that hinge axis orientation requires meticulous consideration during slope‐reducing osteotomies to preserve alignment integrity.

**Level of Evidence:**

Level V, retrospective simulation study.

**Abbreviations**
ACL: anterior cruciate ligament
ACWO: anterior tibial closing wedge osteotomy
CT: computed tomography
CWA: closing wedge angle
FHC: Femoral Head Center
HKA: hip‐knee‐ankle angle
MOWHTO: medial open wedge high tibial osteotomy
MPTA: medial proximal tibial angle
NHA: neutral hinge axis
PCL: posterior cruciate ligament
PTS: posterior tibial slope
SD: standard deviation
TLCP: most posterior point of the lateral tibial plateau
TMCP: most posterior point of the medial tibial plateau
TT: tibial torsion

## INTRODUCTION

The posterior tibial slope (PTS) plays a decisive role in the sagittal stability of the knee joint and is identified as a risk factor in primary anterior cruciate ligament (ACL) and posterior cruciate ligament (PCL) injuries as well as subsequent graft failure [13, 14, 18, 25, 34]. Slope‐changing osteotomies have recently emerged as a surgical treatment option in addressing cases of ACL or PCL injury, particularly in revision cases improving both knee stability and outcomes [1, 3, 5, 18, 28]. While a PTS greater than 12° is often associated with increased ACL injury risk and less than 6° with PCL injuries, these thresholds vary among studies and there is no consensus regarding thresholds for surgical intervention [4, 36].

Anterior tibial closing wedge osteotomy (ACWO) involves a closing wedge osteotomy performed on the anterior aspect of the tibia to reduce the PTS targeting a correction of 5°–10°. In ACWO, the supra‐ and infratuberosity approaches are commonly utilised differing in their effect on patellar height [6].

The relevance of hinge axis orientation in osteotomies, such as in medial open wedge high tibial osteotomies (MOWHTO) for varus deformities show that hinge axis rotation can cause an increased PTS [19, 24, 32]. Studies employing three‐dimensional (3D) analysis and simulation were able to demonstrate the profound impact of hinge axis orientation on anatomical alignment parameters in all three planes [20, 31].

Clinical data suggest that unintended changes in the medial proximal tibial angle (MPTA) occur during slope reducing tibial osteotomies and may be influenced by the size of the preoperative MPTA and the correction angle. In patients with preexisting varus malalignment, an unintended reduction in the MPTA may result in excessive loading of the medial compartment, potentially leading to further joint degeneration. However, the role of hinge axis orientation in ACWO remains unclear to date [23].

The aim of this 3D simulation study is to evaluate the impact of axial and coronal hinge axis rotation on alignment parameters in ACWO. The hypothesis proposes that a NHA in ACWO would prevent alterations in both coronal and axial alignment.

## METHODS

### Study population

This was an institutional review board approved (EC‐Nr. 17‐044) retrospective cohort study. Twenty‐six combined hip, knee and ankle computer tomography (CT) scans of anonymized cases treated for coronal lower limb deformities between 2017 and 2023 as well as post‐mortem whole body CT scans were acquired. Among 1251 CT scans available, a subset of 46 lower limb CT scans was included. The inclusion criteria of an MPTA between 83° and 92° and an HKA between 175° and 183° were selected to create a homogenous study group with near‐neutral frontal lower limb alignment. Exclusion criteria were knee fractures, severe bone defects, knee prostheses and age below 18 or above 50 years. After exclusion of 1205 data sets, a total of 49 CT scans were available for this study. Data sets were categorised into two groups based on their pre‐simulation PTS values: those with a PTS > 12° were assigned to the Slope Group (SG), while those with a PTS ≤ 12° were assigned to the Normal Group (NG).

### 3D anatomy analysis

In all cases, a lower extremity CT scan was conducted in a supine position with a maximum slice thickness of 1 mm. CT data were then used to reconstruct a 3D bone model through Slicer (version 3.5.0, https://www.slicer.org) [8]. An experienced orthopaedic surgeon set the global coordinate system and anatomic landmarks for calculation of PTS, MPTA, TT and HKA of the lower limb in Blender 3.5.1 (Blender Foundation) as previously described [10, 11]. These landmarks already demonstrated a low intra‐ and interobserver variability [10, 11]. To establish the coordinate system, the X‐axis was determined by the best fitting cylinder of both femoral condyles, while the Z‐axis was defined by a line orthogonal to the X‐axis passing through the femoral head center (FHC), consistent with standard practices [9, 21]. Finally, the Y‐axis was defined perpendicular to both the X‐ and Z‐axes. The resulting model enabled an accurate 3D representation of femoral and tibial anatomy. A previous study demonstrated a high agreement between this simulation model and mathematical calculations [33].

### Virtual 3D surgery of ACWO and standard hinge axis

**Figure 1:**
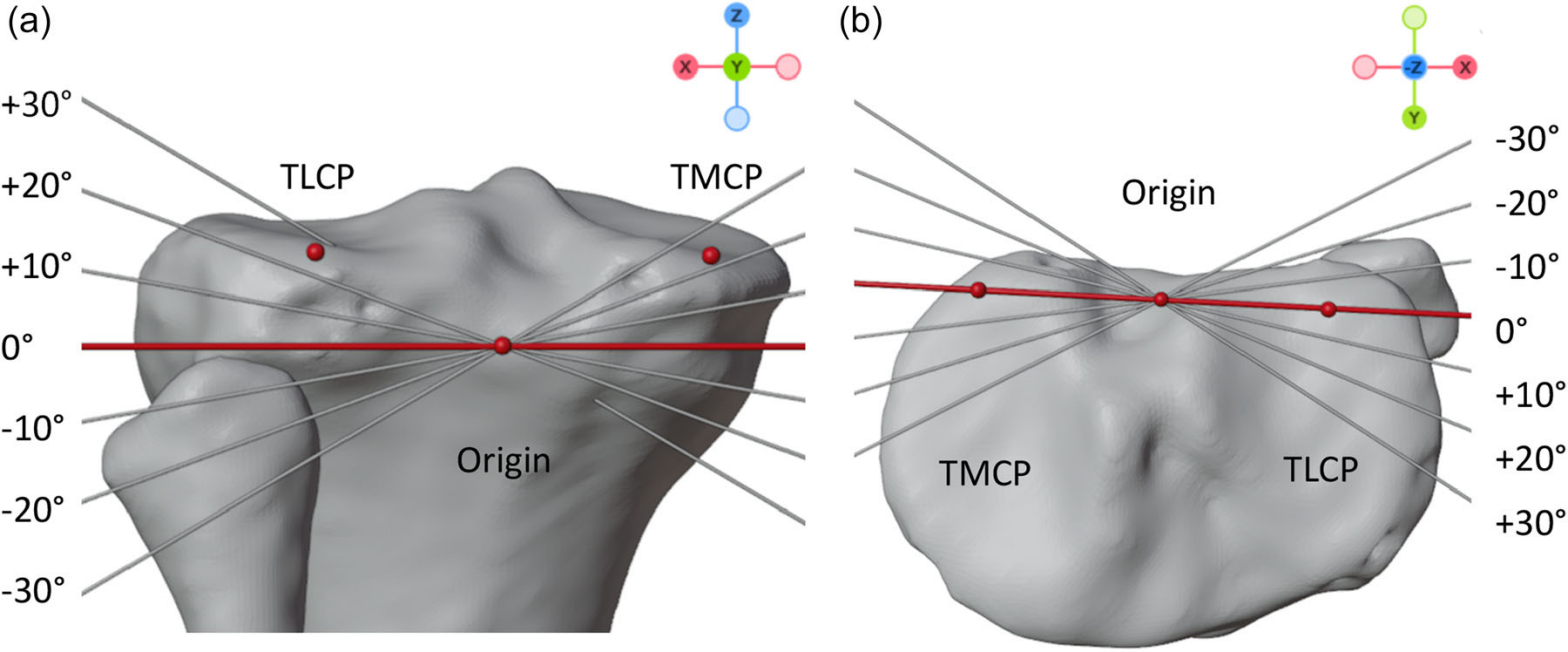
The neutral hinge was oriented parallel to a line passing through the most posterior points of the medial and lateral tibiaplateau (TLCP and TMCP, red points) (red line). An origin (red point) was defined to perform hinge axis rotation. The point is located halfway between TLCP and TMCP and 1 cm underneath the articular surface. Six coronal (a: +: lateral inclined; ‐: medial inclined) and six axial hinge (b: +: anterolateral declined; ‐: anteromedial declined) axes were determined by rotating the NHA at the origin point. NHA, neutral hinge axis; TLCP, most posterior point of the lateral tibial plateau; TMCP, most posterior point of the medial tibial plateau.

A standardised ACWO was performed in the SG as well as the NG virtually using the following method. The current simulation study focuses exclusively on the infratuberosity technique as it reduces the PTS while minimising patellar height alterations and disruption to the tibial tuberosity [29]. The hinge point was calculated as the midpoint of the medial and lateral tibia plateau (TLCP: most posterior point of the lateral tibial plateau, TMCP: most posterior point of the medial tibial plateau) based on predefined landmarks and lowered 1 cm below the articular surface [29]. The position of the resulting hinge point was visually verified to be within the bone model. The hinge axis was oriented parallel to a vector going through TLCP and TMCP line (red line) in the sagittal view and passed through the hinge point as shown in Figure 1 [27].

Thirteen different hinge axes were established, including one NHA, six hinge axes with different orientation in the axial plane (referred to as axial hinge axis), and six hinge axes with different orientation in the coronal plane (referred to as coronal hinge axis). The NHA was characterised as a sagittal and axial rotation of 0° degrees (Figure 1). The NHA minimises alignment changes during ACWO because it is orthogonal to the tibial plateau in the frontal view, preventing MPTA alterations, and parallel to the posterior tibial plateau in the axial view, maintaining tibial torsion. Additionally, six axial hinge axes (±10°, ±20° and ±30°) were generated by rotating the NHA in the axial view (as depicted in Figure 1). A positive value indicated that the hinge axis was rotated anterolaterally on the axial plane, while a negative value indicated a posterolateral rotation. Similarly, six coronal hinge axes (±10°, ±20° and ±30°) were created by rotating the NHA around the hinge point in the sagittal plane (as illustrated in Figure 1). A positive value represented a lateral‐inclined orientation in the coronal plane, while a negative value indicated a medial‐inclined orientation in the coronal plane of the coronal hinge axis.

**Figure 2:**
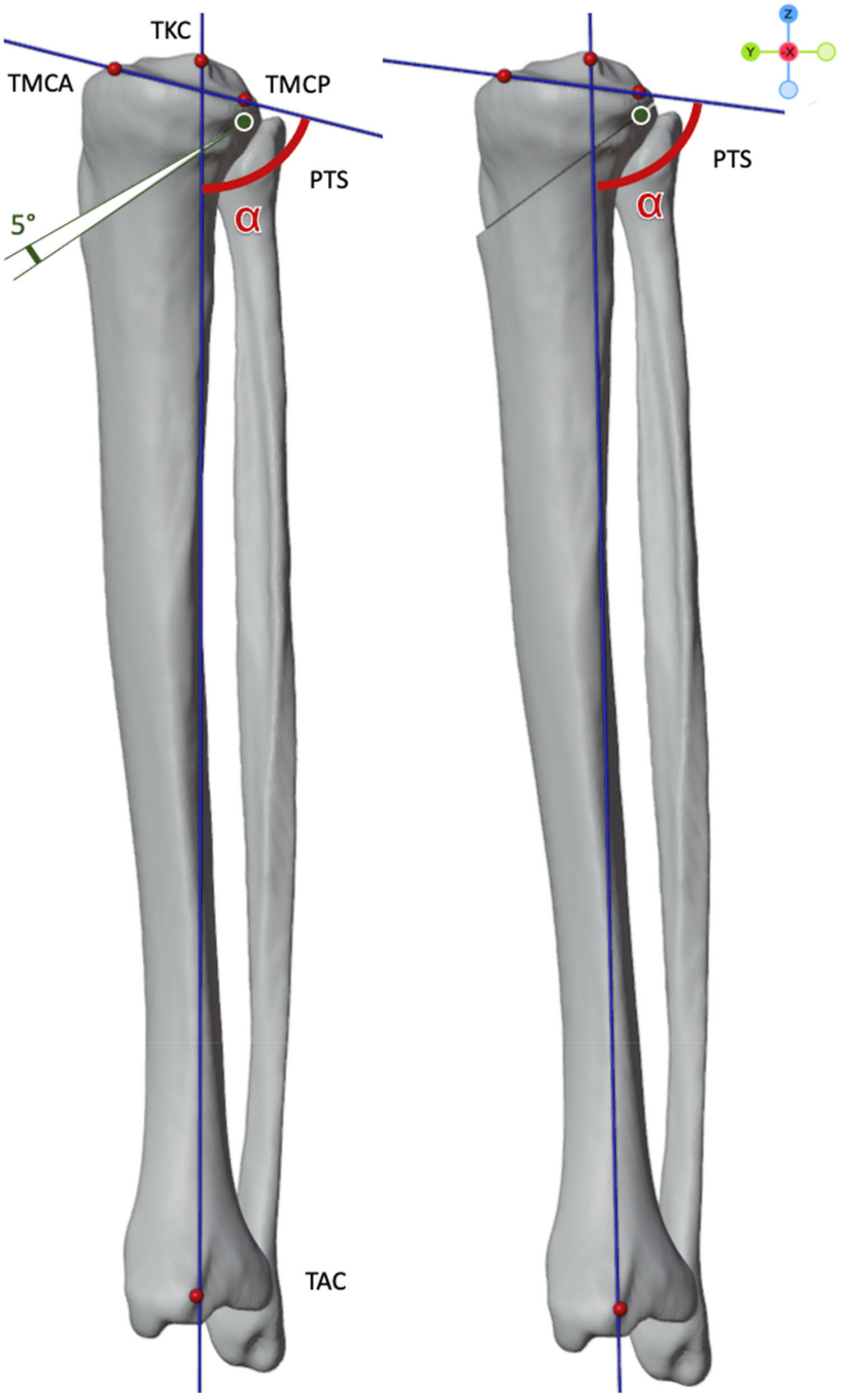
Sagittal view on the simulation of an anterior closing wedge tibial osteotomy performed with an 5° closing wedge angel (CWA) and rotation around the neutral hinge axis (green point). The posterior tibial slope (PTS) is measured by the angle of the tibial anatomic axis (connecting line between the knee (TKC) and ankle center (TAC)) and a line connecting the anterior (TMCA) and posterior (TMCP) point of the medial tibial plateau.

The osteotomies were simulated by incrementally increasing the closing wedge angle (CWA) in one‐degree steps, from 1° to 5° for each hinge axis, resulting in a total of 3185 simulations (Figure 2).

### Outcome measurements

Using anatomical landmarks with proven excellent inter‐ and intra‐rater reliability, outcome measurements were obtained both pre‐ and post‐simulation through a custom Python script implemented in Blender: PTS, tibial torsion (TT), MPTA and HKA [10, 11]. PTS was defined as the angle between the medial tibia plateau joint line and the tibial anatomical axis in the sagittal plane as shown in Figure 2 [7, 35]. TT was measured according to Goutallier in the axial view [12]. For each simulation, the PTS, MPTA, HKA and TT were measured in the 3D bone models. To evaluate the influence of different hinge axes, outcome measurements were analysed following an ACWO with a 5° closing wedge angle (CWA) [29].

### Statistical analysis

All data were given as mean value and standard deviation, and, if normal distribution was not given, as mean, first and third quantile. The sample size was calculated using G*Power 3.1.9.6 based on mean change of the MPTA (1.3° ± 1.5°) in anterior tibial closing wedge osteotomy in a previous study [23]. A minimum sample size of 9 was required to achieve a Power of 80% (effect size = 0.87, *α* = 0.05).

Normal distribution was tested using the Shapiro–Wilk test. After confirming non‐normal distribution, the distribution variation between groups was determined by the Kruskal–Wallis test. Post hoc testing via pairwise Wilcoxon test was used to assess differences between two dependent samples. Pearson's correlation coefficients (*r*) were used to assess the extent to which two variables are related: 0.1 < *r* < 0.3 indicating weak correlation, 0.3 < *r* < 0.7 indicating moderate correlation, and 0.7 < *r* < 1.0 indicating strong correlation. The significance level was set at 0.05. The analysis was performed in R Studio Version 2023.03.1 (Posit Software, PBC).

## RESULTS

### Pre‐simulation alignment in the slope group and the normal group

Forty‐eight cases were anonymously included in this study. Demographic data are displayed in Table 1.

**Table 1:** Pre‐simulation values of the valgus and normal alignment group.

	Slope group (*n* = 21)		Normal group (*n* = 28)		
	Mean	SD	Mean	SD	*p* value
PTS [°]	15.0	2.2	8.9	2.2	<0.001***
Tibial torsion [°]	34.8	9.2	37.1	7.8	>0.05
MPTA [°]	87.2	1.6	88.1	2.4	>0.05
HKA [°]	178.0	1.5	179.0	2.1	>0.05

Abbreviations: HKA, hip‐knee‐ankle‐angle; PTS, posterior tibial slope; MPTA, mechanical proximal tibial angle; SD, standard deviation.

### Changes of alignment parameters by 5° CWA ACWO

After an ACWO of 5° CWA in the NHA, the TT, MPTA and HKA showed no significant changes (Table 2).

**Table 2:** Mean change of alignment parameters in comparison to pre‐simulation parameters (*n* = 49).

	Mean	SD	*p* value
PTS [°]	−10.79	1.1	<0.001
TT [°]	−2.4	8.5	0.534
MPTA [°]	+0.01	0.03	0.935
HKA [°]	+0.01	0.03	0.932

Abbreviations: HKA, hip‐knee‐ankle‐angle; PTS, posterior tibial slope; MPTA, mechanical proximal tibial angle; SD, standard deviation; TT, tibial torsion.

### Medial proximal tibia angle

**Figure 3:**
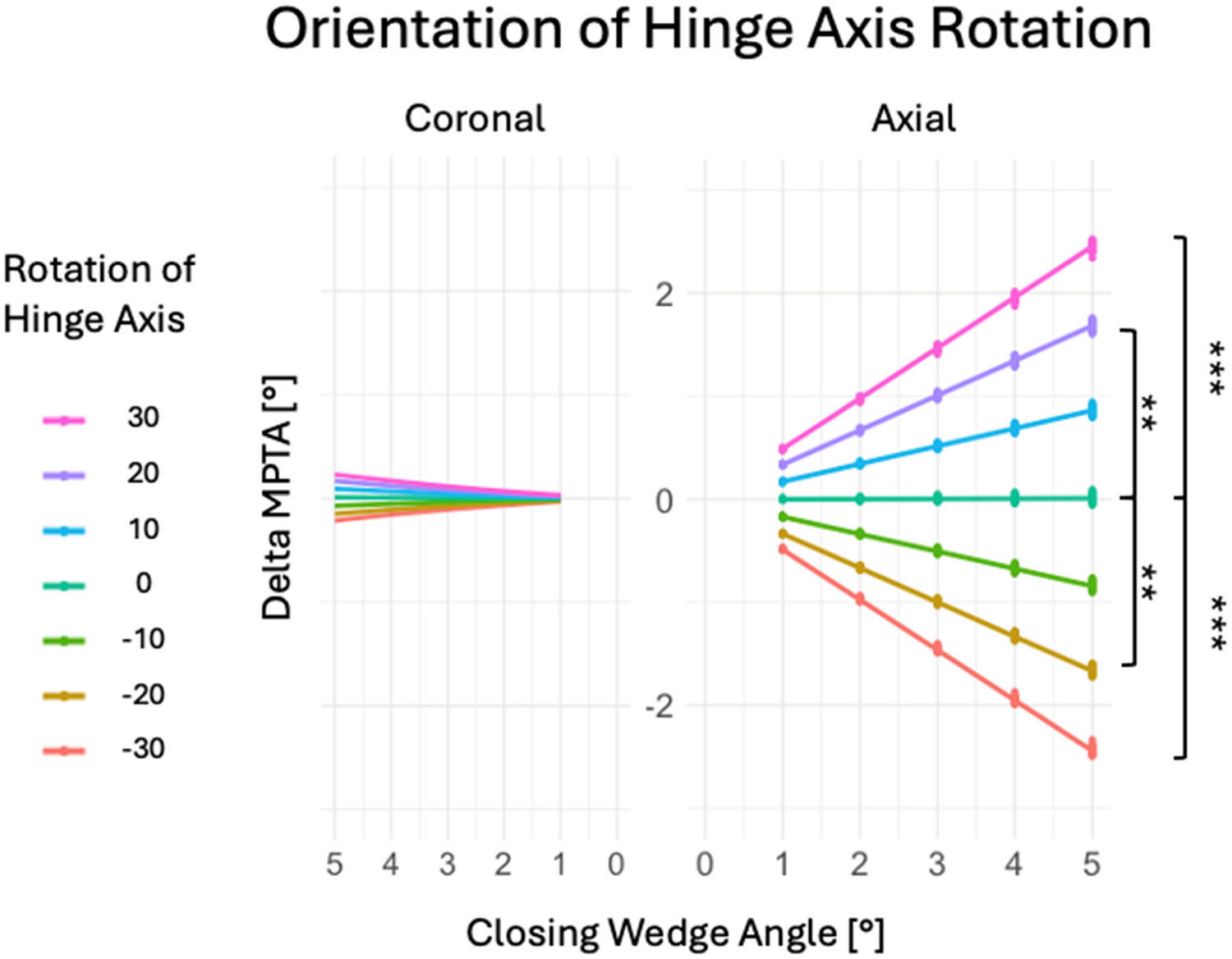
Axial rotation of the hinge axis significantly influences MPTA changes compared to the NHA with a linear relationship to the closing wedge angle (CWA). Coronal hinge axis rotation did not significantly influence MPTA changes. Each coloured lines indicate the linear regression trends for each rotation of the hinge axis. Significance levels are indicated by brackets and asterisks: **p < 0.01, ***p < 0.001. MPTA, mechanical proximal tibial angel; NHA, neutral hinge axis.

**Figure 4:**
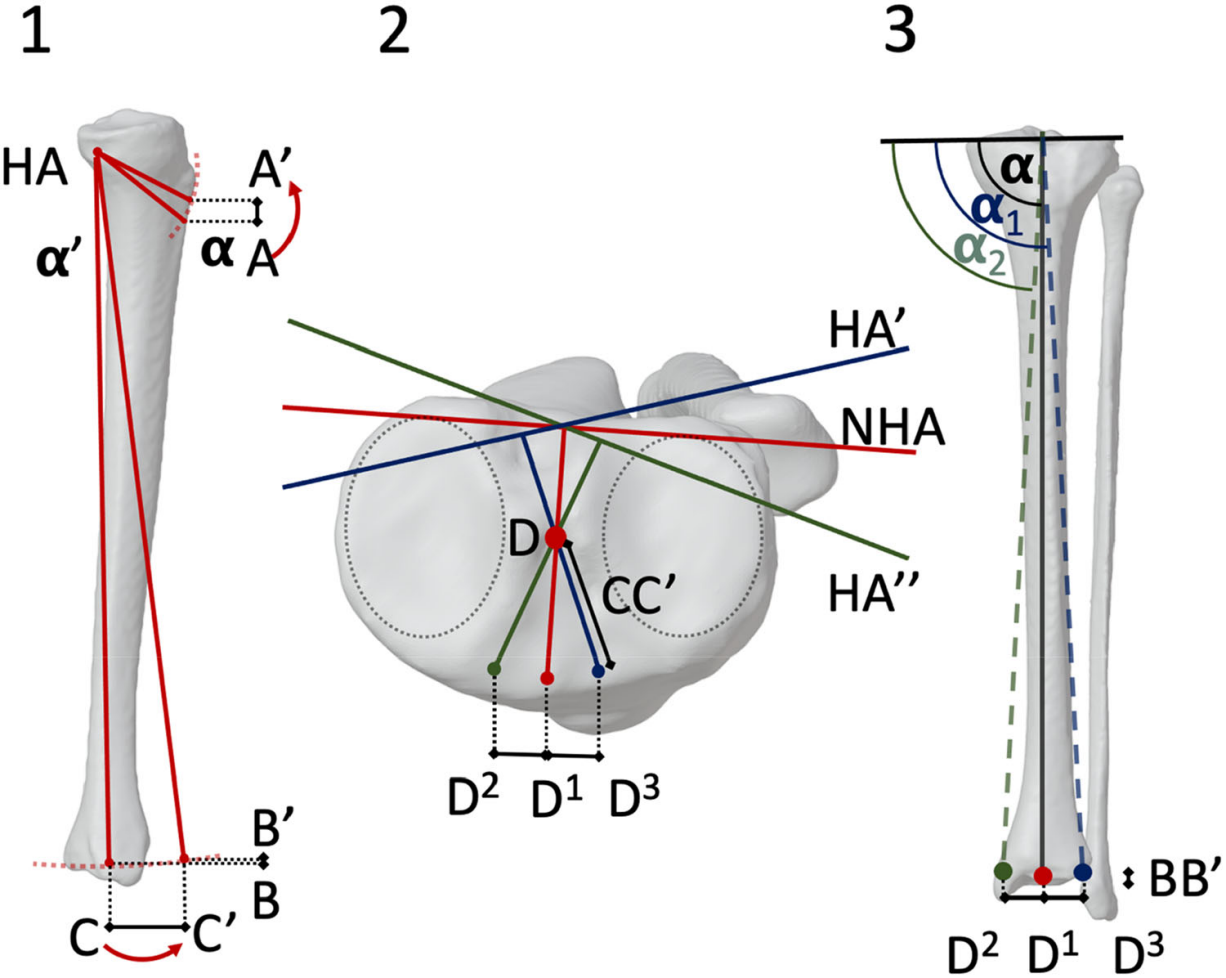
Impact of hinge axis rotation during anterior tibial closing wedge tibial osteotomy (ACWO) in the axial plane on the mechanical proximal tibia angle (MPTA) of a left knee. The neutral hinge axis (NHA) is oriented parallel to the posterior tibial plateau.

**Figure 5:**
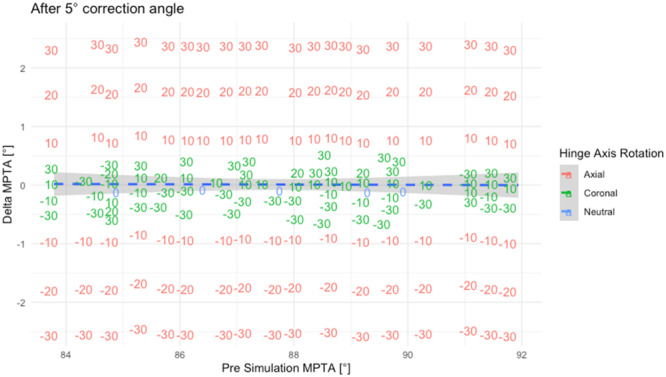
Hinge axis rotation in the axial plane leads to changes of the medial proximal tibial angle (MPTA). Change of the MPTA did not correlate with the pre‐simulation MPTA. Datapoints are displayed as the value of hinge axis rotation in red for axial and green for coronal rotation. A correlating line is displayed in blue showed no significant correlation (*p* = 0.9). Overlapping datapoints were deleted to increase readability.

Axial hinge axis rotation significantly affected the MPTA in ACWO with a 5° CWA (*p* < 0.001). Anteromedial rotations exceeding 10° increased MPTA, while anterolateral rotations greater than 10° decreased MPTA siginificantly (Table 3 and Figure 3). Each 10° axial rotation caused a linear MPTA change of 0.86° ± 0.03°, as explained by a geometric model (Figure 4). Pre‐simulation MPTA did not correlate with MPTA changes (*r* < −0.01, *p* > 0.05; Figure 5).

(1) During closure of the ACWO by an angle of **α**°, the osteotomy entry point A shifts to A', the coronal ankle joint position B shifts to B', and the sagittal ankle joint position C shifts to C', following a circular trajectory. This movement results in a significant change in the sagittal position of the ankle center (CC'), while only minimal translation occurs along the coronal axis (BB'). Conversely, the osteotomy entry point experiences a more substantial displacement along the coronal axis (AA'), which is a multiple of the BB' distance.
(2) Considering various hinge axes (NHA, anteromedially rotated hinge axis (HA'), and anterolaterally rotated hinge axis (HA'')) in the axial view, the displacement of the axial ankle joint center (D) by the CC' distance, perpendicular to the hinge axis, leads to either medialization (D^2^) or lateralisation (D^3^) of the ankle joint.
(3) In the coronal view, the effect of different hinge axis rotations on coronal alignment is depicted. An anteromedial hinge axis rotation (HA') results in lateralisation of the ankle joint (D^3^) and an increase in the mechanical proximal tibial angle (MPTA). In contrast, an anterolateral hinge axis rotation (HA'') causes medialization of the ankle (D^2^) and a corresponding decrease in the MPTA. The neutral hinge axis (NHA) has minimal influence on the coronal position of the ankle center (D^1^), thereby preserving the initial MPTA.

**Table 3:** Mean change of the MPTA and PTS following ACWO with a 5° CWA correction.

Hinge parameter		Mean MPTA change [°]	SD of mean MPTA change	*p* value	Mean delta slope	Mean SD delta slope	*p* value
Orientation of hinge axis rotation	Magnitude of hinge axis rotation [°]						
Group differences		<0.001***			<0.001***		
Axial	−30	−2.4	0.03	<0.001***	−0.2	0.56	1.0
	−20	−1.7	0.03	0.01**	0.2	0.38	1.0
	−10	−0.8	0.03	>0.05	0.3	0.2	1.0
	+10	0.9	0.03	>0.05	−0.6	0.2	1.0
	+20	1.7	0.03	0.01**	−1.5	0.41	1.0
	+30	2.5	0.03	<0.001***	−2.7	0.62	0.02*
Coronal	−30	−0.2	0.21	>0.05	−1.3	0.28	1.0
	−20	−0.1	0.15	>0.05	−0.5	0.19	1.0
	−10	−0.1	0.08	>0.05	−0.1	0.1	1.0
	+10	0.1	0.08	>0.05	−0.2	0.11	1.0
	+20	0.2	0.15	>0.05	−0.8	0.23	1.0
	+30	0.2	0.22	>0.05	−1.7	0.36	0.6

*Note*: The mean changes in MPTA and PTS were calculated by determining the average difference between values obtained after ACWO with a closing wedge angle (CWA) of 5° using various hinge axes, compared to the neutral hinge axis (NHA). Differences in MPTA and PTS between the various hinge axis orientations and the NHA were statistically analysed for both overall group differences and individual pairwise comparisons.

Abbreviations: ACWO, anterior tibial closing wedge osteotomy; CWA, closing wedge angel; MPTA, mechanical proximal tibial angel; PTS, posterior tibial slope.

### Posterior tibial slope

**Figure 6:**
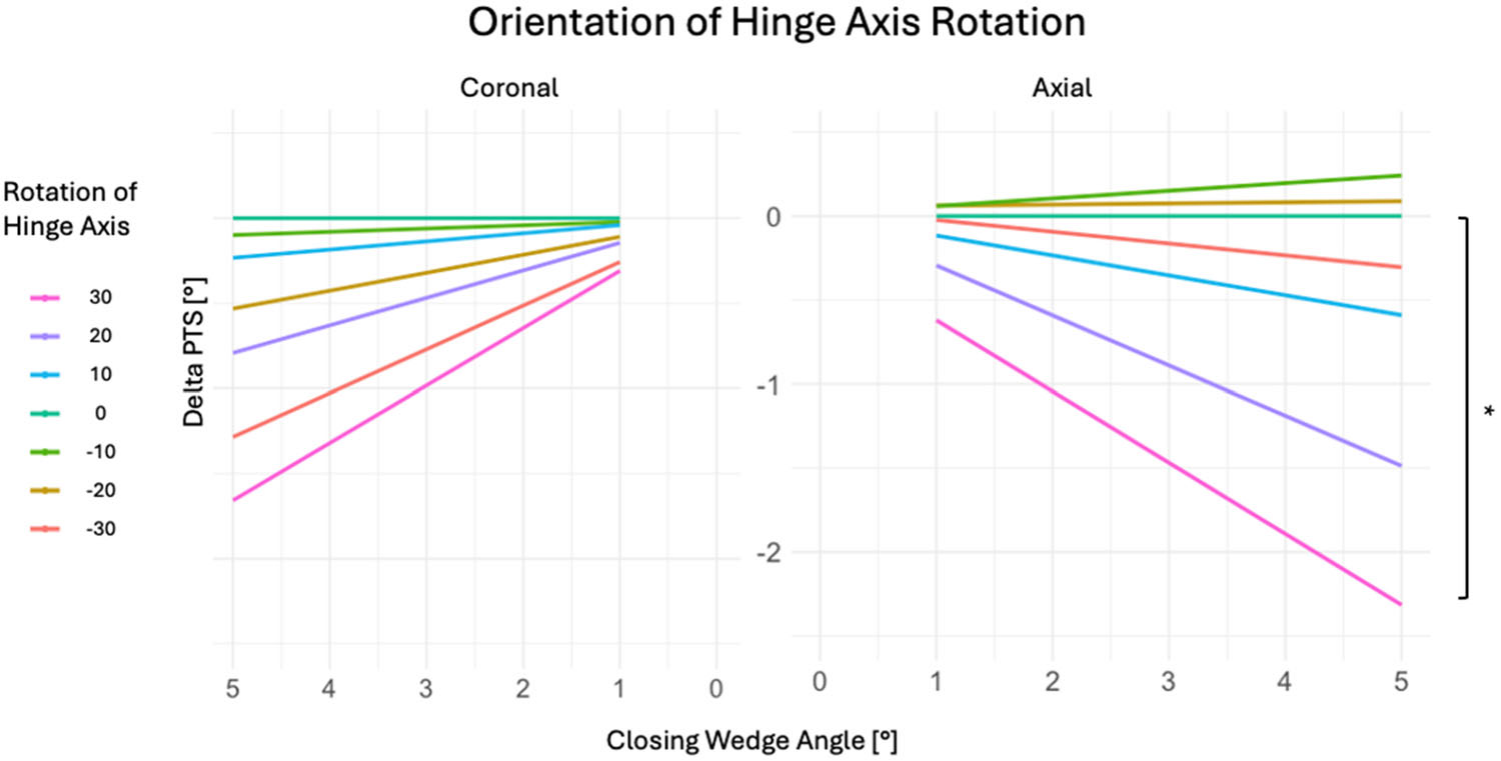
Coronal as well as axial hinge axis rotation tend to decrease PTS change after ACWO with a CWA of 5° compared to NHA. Each coloured lines indicate the linear regression trends for each rotation of hinge axis. Significance levels are indicated by brackets and asterisks: *p < 0.05. ACWO, anterior tibial closing wedge osteotomy; CWA, closing wedge angel; PTS, posterior tibial slope.

Hinge axis rotation in ACWO tended to result in a smaller change in PTS compared to the NHA. Significant group differences were identified (*p* < 0.001, Table 3, Figure 6), Pre‐simulation PTS did not correlate with PTS change (*r* < −0.01, *p* > 0.05).

### Tibial torsion

Neither axial nor coronal changes of the hinge axis led to significant changes of the tibial torsion (*p* > 0.05).

## DISCUSSION

The main finding was that the MPTA is altered significantly in ACWO when the axial hinge axis rotation exceeds 10°. Notably, coronal rotation did not significantly impact the MPTA. Furthermore, the defined NHA in ACWO did not lead to changes in the coronal or axial tibial alignment. The NHA is parallel oriented to the posterior tibial plateau and placed 1 cm underneath the articular surface. There was a tendency towards a decrease in PTS change following ACWO when hinge axis rotation was applied.

Clinical data suggest that ACWO can significantly reduce MPTA following slope correction. In a cohort of 48 patients who underwent infratuberosity ACWO, Weiler et al. [34] reported a non‐significant MPTA decrease of 1.1° using both infra‐ and supratuberosity approaches. Similarly, Cance et al. [2] found a reduction of 0.95° (confidence interval 95% 0.44°–1.46°) with the supratuberosity technique, while Mayer et al. [23] described a decrease of 1.3° ± 1.5° in 38 patients using the infratuberosity approach. In the supratuberosity approach, Luceri et al. [22] measured the coronal alignment based on the medial tibial plateau–tibial shaft angle (mTPTS) and noticed a higher varus alignment from 0.3° ± 3.0° preoperatively to 2.4° ± 2.1° postoperatively (not significant) in a series of 12 cases. No MPTA alteration were observed after ACWO performed in a NHA in this simulation study. Additionally, Mayer et al. [23] identified a lower preoperative MPTA as a predictive factor for MPTA decrease, but there was no correlation between initial MPTA and MPTA change when ACWO was performed with the NHA in this model. In this simulation study, axial hinge axis rotation emerges as the most influential factor for postoperative MPTA change.

The systematic changes in MPTA during ACWO can be explained using a geometric model of the tibia in all three planes. Axial rotation of the hinge axis during ACWO affects the MPTA by causing mediolateral shifts of the ankle joint position. Using the NHA, oriented parallel to the posterior tibial plateau, significant sagittal displacement of the ankle center occurs with minimal coronal translation, preserving the initial MPTA. However, rotating the hinge axis anteromedially leads to lateralisation of the ankle joint and an increase in MPTA, while an anterolateral rotation causes medialization and a decrease in MPTA. Thus, the rotation of the hinge axis in the axial plane directly influences coronal alignment during ACWO: anteromedial rotation increases MPTA due to lateral ankle shift, and anterolateral rotation decreases MPTA due to medial ankle shift. Utilising the NHA minimises these coronal positional changes, helping to maintain the original MPTA.

It was hypothesised, that unintentional MPTA changes originate from anatomic tibial morphology and the operational technique. The presence of the proximal tibiofibular joint as well as the triangular shape of the tibia can cause difficulties in the approach of the posterolateral aspect and may lead to incomplete osteotomies [26]. This could lead to asymmetrical closure affecting the coronal alignment. Finally, a plate‐fixation method is suspected to alter the postoperative MPTA by applying more compression to the medial side. When using staples for fixation, it is recommended to place the lateral staple first to maximise lateral compression and avoid iatrogenic varus [2].

To achieve an optimal aligned hinge axis intraoperatively, the hinge axis should be oriented parallel to the posterior tibiaplateau. Intraoperative fluoroscopy adjustments to position the knee in a true anterior‐posterior position of the tibia, as proposed by Mayer et al. [23], can aid in this process. Relying solely on the orientation of the femoral condyles intraoperatively may introduce errors, particularly in knees with high intraarticular tibiofemoral version [17]. Additionally, surgeons should ensure cutting from the anterior perpendicular to the hinge axis and carefully close the osteotomy not only on the medial side where plate positioning is typically performed. Intraoperative orientation from the anteromedial aspect caused by the surgeon's position or medial side compression during plating could potentially decreasing the MPTA.

The planned hinge axis can be altered during surgery by factors such as soft tissue constraints, fixation methods, and bony morphology, resulting in a true hinge axis that deviates from the intended orientation. The effect of this alteration can be determined using 3D simulation. 3D simulation shows significant advantages compared to conventional biomechanical studies regarding quantity, precision and reliability [16, 30].

## LIMITATIONS

This study has several limitations. First, the 3D simulations did not account for soft tissue structures, which may influence alignment outcomes in vivo. Additionally, the models were generated in a non‐weight‐bearing position, limiting the accuracy of alignment changes under physiological loads [15]. The focus on the infratuberosity technique and the availability of only 49 data sets restrict the generalisability of the findings and comparability to other ACWO approaches and cut positioning, length, and sagittal incline may vary substantially between techniques. Small changes in MPTA observed in the study may not be clinically significant in terms of patients' outcome. Lastly, the simulations do not fully replicate intraoperative variability, such as hinge axis misalignment or incomplete osteotomy, which may impact surgical results.

## CONCLUSION

In conclusion, axial hinge axis rotation significantly affects the coronal alignment during ACWO and could potentially explain unintended changes of the MPTA. A neutral hinge, parallel to the posterior part of the medial and lateral tibia plateau, does not change coronal lower limb alignment. By understanding the geometrical principles underlying PTS correction, postoperative alignment can be optimised in the management of complex knee pathologies.

## AUTHOR CONTRIBUTIONS

Julian Fürmetz and Julius Watrinet conceived and planned the experiments. Julius Watrinet and Philipp Blum carried out the simulations. Markus Bormann contributed to sample preparation. Julian Fürmetz, Sebastian Siebenlist and Boris Holzapfel contributed to the interpretation of the results. Julian Fürmetz and Julius Watrinet took the lead in writing the manuscript. All authors provided critical feedback and helped shape the research, analysis and manuscript. Daniel Berger contributed to this work by sample preparation.

## CONFLICT OF INTEREST STATEMENT

The authors declare no conflicts of interest.

## ETHICS STATEMENT

The study was approved by the local ethics committee (NR 17‐044, 2017 Munich). This is a retrospective study. All patient information was deidentified and patient consent was not required.

## Data Availability

Data is not available.
